# Obstacles in the road of biomedical research on COVID-19 in Jordan: Poor funding and beyond

**DOI:** 10.7189/jogh.12.03052

**Published:** 2022-07-16

**Authors:** Arwa Qaqish, Mariam Al-Omari, Rana Dajani

**Affiliations:** 1 Department of Biology and Biotechnology, Faculty of Science, Hashemite University, Zarqa, Jordan; 2 Department of Basic Sciences Faculty of Medicine, Yarmouk University, Irbid, Jordan

## BIOMEDICAL RESEARCH ON COVID-19, A BURST OF INVESTIGATIONS AND A LOT OF WASTE

Since the spread of COVID-19, dedicated researchers from all over the world worked round the clock to unveil the identity of the new virus and find ways to bring the deadly pandemic to an end. Basic science virologists revealed facts about the viral life cycle, cell entry, replication, and emerging variants. Epidemiologists carefully tracked the disease’s transmission dynamics. Immunologists studied detailed immune response to infection. Pharmacologists performed drug design and drug repurposing studies. Biotechnologists designed and developed vaccines and efficient diagnostic techniques. Physicians and pathologists investigated symptoms and pathogenesis of infection and developed therapeutic management strategies to help rescue critical cases. In addition, many case reports and clinical trials have been performed, and many other social and economic aspects of the pandemic have been studied.

The flood of research articles related to COVID-19 has been very difficult to track with thousands of articles being published at an accelerated rate in a short time. By December 20, 2021, in terms of basic science and medical research, searching “COVID-19” at PubMed returns 211 708 publications, while “SARS CoV-2” returns 133 073 results [1].

This breathtaking speed of the medical research response to the pandemic has come with mixed consequences. On the positive side, there has been a greater provision of open access to COVID-19 studies, increased institutional collaboration, expedited ethical approvals of new studies, and prompter use of preprints. On the negative side, time pressure, along with inadequate infrastructure, amplified the problems of research waste due to poor questions, design and performance, lack of or poor reporting of results, and other redundancies [2].

## BIOMEDICAL RESEARCH ON COVID-19 IN JORDAN IS MAINLY CONFINED TO SURVEY-BASED STUDIES

Through a simple search, we investigated the contribution of Jordanian scientists to COVID-19 biomedical research. Based on PubMed, there are 392 articles published by researchers from Jordan from the beginning of the pandemic to December 20, 2021. Interestingly, 216 (54.1%) of these articles are questionnaire-based and meta-analysis studies including cross-sectional, longitudinal, and qualitative investigations. The rest are divided into review articles (n = 71, 18.1%), informatics analysis and mathematical modelling (n = 52 13.3%), reports and commentaries (n = 24, 6.1%), retrospective studies (n = 23, 5.9%), database studies (n = 21.0%) and letters to editors (n = 2, 0.5%). Only 3 (0.8%) of research papers performed simple kit-based work to measure serum antibody levels to determine antibody titers in the context of natural infection and vaccination. Unfortunately, there is not a single hard-core virology or immunology laboratory based experimental investigation or even a clinical trial. Additionally, publication redundancy was evident; there are over 20 articles investigating vaccine hesitancy in Jordan alone or among other countries [3].

### Poor funding is the main obstacle faced by biomedical research on COVID-19 in Jordan

For a developing country with limited resources such as Jordan, research would not be prioritized if a pandemic threatened lives and the economy. Any available funding would certainly be used for directly controlling disease spread through improving diagnosis and treatment strategies. For example, in the beginning of 2021, the Jordanian Government hugely expanded the ministry of health’s molecular diagnosis capacity to involve every governorate and travel border, to help better manage the transmission of COVID-19 [4]. Another priority was to establish platforms for online school and college education [5,6]. The government also helped those who lost their jobs because of the pandemic to secure food and other basic needs [7].

Still, this does not rule out the benefits of investing funds in proper hard-core biomedical research in the country. On the long run, this could lead to the production of local diagnostic kits and vaccines and help flourish the country economically.

Several reasons lie behind the obvious imbalance in types of articles, research topics and research tools used in Jordan. The enormous number of survey-based studies and review articles reflect the scarcity of resources among other potential barriers. Other barriers are lack of equipment to perform whole genome sequencing if we want to understand not only the virus but the variation in the human host and variation in susceptibility to infection.

A very concrete obstacle for Jordan’s COVID-19 biomedical research is the lack of a biosafety level 3 (BSL-3) facility. Due to its high infectivity and pathogenicity, SARS CoV-2 should be handled in BSL-3 facilities, designed for work involving microbes which can cause serious and potentially lethal disease via inhalation. BSL-3 facilities are very expensive to construct and maintain. Workers in such facilities should go through extensive training and follow very strict protocols to keep pathogens contained and not cause their spread to the outside environment [8]. To the best of our knowledge, there is no such facility dedicated to virus research available for researchers in Jordan. This forms a significant barrier for developing national anti-viral candidate vaccines and therapeutics.

The lack of proper biomedical research seems to be caused by more than the absence of a BSL-3 facility for handling, propagating, and storing native virus in Jordan. In search for other options where native infective SARS-CoV-2 cannot be used, pseudoviruses are safe alternatives that proved to very useful for viral entry studies [9].

**Figure:**
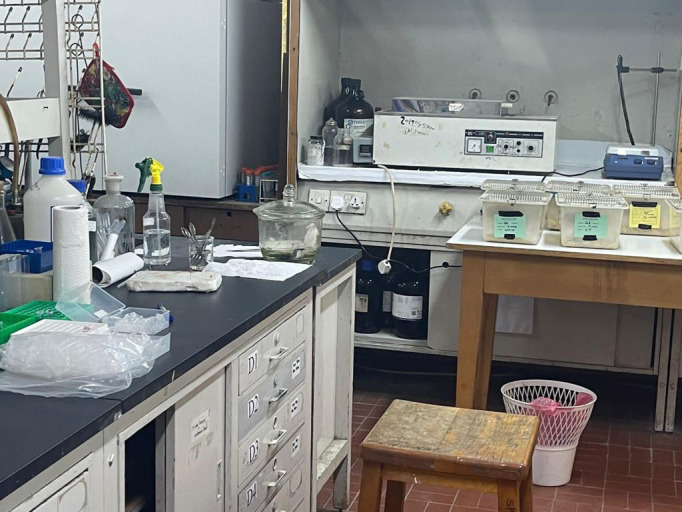
Photo: The Immuno-Parasitology Research Laboratory, at the Department of Biology, Yarmouk University, Irbid, Jordan. Source: from the authors’ collection, used with permission.

Viral pseudotyped particles, or pseudoviruses are enveloped virus particles, typically derived from retroviruses or rhabdoviruses, that harbor heterologous envelope glycoproteins on their surface and a genome lacking essential genes, mainly genes important for viral replication. These synthetic viral particles are safer surrogates of native viruses and acquire the tropism and host entry pathway characteristics governed by the heterologous envelope glycoprotein used [9,10]. They have proven to be very useful tools used in research with many applications, such as enabling the study of infection and entry mechanisms of enveloped viruses, quantification of anti-virus neutralizing antibodies and screening of potential viral inhibitors. Moreover, pseudoviruses can be designed as vaccine candidates to protect against native virus infection [10].

No pseudovirus-based studies are being or have been performed in Jordan. To our knowledge, there are no basic virology laboratories where viruses are being studied, produced and stored in the country’s academic institutions [11]. Despite the remarkably few numbers of basic virologists and immunologists in Jordanian universities and research centers, we believe that the problem of lack of basic virology and immunology research in the country is more than lack of facilities and resources.

## BEYOND FUNDING, BUREAUCRACY STANDS IN THE ROAD OF PROPER BIOMEDICAL RESEARCH IN JORDAN

As reported by a survey study performed by Al-Fanar Media, poor funding was the main obstacle faced by Arab researchers among a complex web of other barriers [12]. Administrative obstacles, delayed approvals, restrictions on the cross-border transportation of samples and equipment, and difficulty in international travels and collaborations are challenges common to Jordan and Arab countries. For Jordan, university regulations for rank-to-rank promotions form a solid barrier for developing good quality research, especially in the basic sciences and biomedical fields. As reported by Dr Dajani of the Hashemite University of Jordan: “Many are stuck inside a loop of wanting to be promoted and getting a better salary, and that’s it. The objective becomes promotion, not doing science, so they do the bare minimum to get promoted,”. On a personal note, based on university promotion regulations, academic professors get higher promotion points based on the number of publications, regardless of the quality of the research. Moreover, the 1-to-2-year period given to new professors to pass from contract-based employment to tenure track does not take into consideration establishing laboratories and the time needed to perform experiments. This leads them to search for more time efficient alternatives of writing reviews and performing questionnaire-based studies in fear of losing their jobs or their promotion being delayed. Also, all universities of Jordan require full time teaching from new academic members without considering the time needed for research.

## CONCLUSIONS

There is a tremendous lack of proper, investigatory, and experimental biomedical research conducted in research laboratories in Jordan. In the COVID-19 era, Jordanian biomedical scientists were willing to help the globe end the pandemic but felt tied and helpless. Whether establishing well-equipped research laboratories at higher biosafety levels is a priority to an economically weak country is questionable. However, poor funding is not the only problem faced by scientists in Jordan, as bureaucracy is standing in the way.

Another important point is the lack of collaboration between neighboring Arab countries that share the same history, culture, ethnicity, and environment. Such collaboration could create an opportunity to coordinate efforts and share learnings. Unfortunately, politics prevent such collaborations. Thus, this is a call to scientists across the region to reach out and build ties across universities and research institutions to spark the start of such endeavors that will have long-term payoffs for containing the virus and preventing further spread.
